# *De Novo* Mutation of Paternal *IGF2* Gene Causing Silver–Russell Syndrome in a Sporadic Patient

**DOI:** 10.3389/fgene.2017.00105

**Published:** 2017-08-08

**Authors:** Deguo Liu, Yajian Wang, Xiu-An Yang, Deyun Liu

**Affiliations:** ^1^Department of Paediatrics, The Second Hospital of Anhui Medical University Hefei, China; ^2^Joy Orient Translational Medicine Research Center Co., Ltd. Beijing, China; ^3^Beijing Scientific Operation Biotechnology Co., Ltd. Beijing, China

**Keywords:** *de novo* mutation, IGF2, Silver–Russell syndrome, growth retardation, sporadic patient

## Abstract

Silver–Russell syndrome (SRS) is a rare, but well-recognized disease characterized by growth disorder. To date, there are two reports arguing *IGF2* mutation for the onset of SRS. Herein, we present another sporadic case harboring *IGF2* mutation. The male proband was the first and only child of a non-consanguineous Chinese couple. He was small for gestational age, with relative macrocephaly at birth. Severe feeding difficulties, low feeding, and growth retardation were revealed during neonatal period. At 4.5 years old, obvious body asymmetry was noted. Whole exome sequencing identified a novel *de novo* c.101G > A (p.Gly34Asp, NM_000612) variant in *IGF2* and Sanger sequencing validated the variant. Amplification refractory mutation system polymerase chain reaction demonstrated that the *IGF2* variant was on the paternal allele. Alignment shows the variant is evolutionarily conserved. Structural modeling argues that the variant site might be important for the binding of IGF2 to its receptor. Our study provides further evidence that IGF2 mutation may be another mechanism of SRS, and we consider that IGF2 should be included in a disease specific gene panel in case it is designed for SRS routine diagnostics.

## Introduction

Silver–Russell syndrome (SRS) is a rare disorder found in children with low birth weight, postnatal short stature, characteristic facial features, and body asymmetry (Silver et al., 1953; Russell, 1954). The etiology and clinical characteristics of SRS are extremely heterogeneous. Loss of methylation on chromosome 11p15 and maternal uniparental disomy for chromosome 7 are considered as the most common molecular pathology. Azzi et al. (2015) developed the only scoring system (Netchine–Harbison scoring system) using prospective data, which, is helpful to SRS clinical diagnosis This system includes clinical manifestations of small for gestational age, postnatal growth failure, relative macrocephaly at birth, prominent forehead, body asymmetry, and feeding difficulties. For molecular diagnosis, numerous copy number variants and DNA methylation analysis involving 11p15.5 region and chromosome 7 are mostly recommended [4]. Additionally, tests of chromosome 14q32 variants, *CDKN1C* mutations, and multi-locus imprinting disturbance are also suggested (Wakeling et al., 2017).

Begemann et al. (2015) identified *IGF2* variant (c.191C > A, p.Ser64Ter) in a multigenerational family with four members presenting growth restriction. As no report regarding SRS caused by *IGF2* mutation had been published, the authors are not sure about the contribution of IGF2 to prenatal and postnatal growth. In another study, the same authors argue that *IGF2* mutation analysis is not indicated in sporadic SRS cases (Muller et al., 2016). Recently, a Japanese team reported the first case of *de novo IGF2* mutation in a patient with SRS, arguing that IGF2 mutation analysis is helpful in SRS patients negative for other etiologies (Yamoto et al., 2017). In line with this, the obscure relationship between *IGF2* mutation and SRS becomes clear. Herein, we present a sporadic case of SRS with *de novo* variant in *IGF2* to expand the molecular and phenotypic spectrum of *IGF2* mutations induced SRS.

## Case Presentation

**FIGURE 1:**
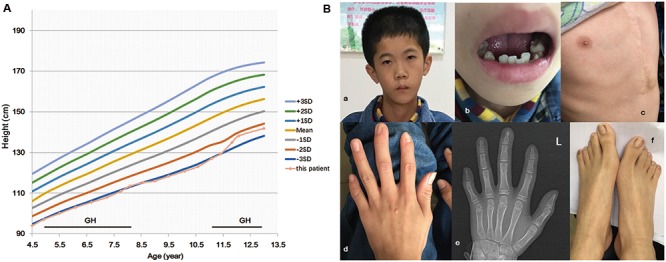
IGF2 mutation c.101G > A (p.G34D) in a patient with features of the Silver–Russell syndrome. Growth chart of the patient **(A)**. Period of human growth hormone (GH) therapy is indicated by the horizontal bar. The patient has facial dysmorphisms of triangular face, micrognathia, low-set ears **(Ba)**, and irregular teeth **(Bb)**. He had taken surgical treatment due to atrial septal defect **(Bc)**. Finger and toe deformities were revealed **(Bd–f)**.

The proband was a 13-year-old boy who was the first and only child of a non-consanguineous Chinese family. The parents were clinically normal and no members presented a developmental delay in his family. He was delivered at 37 weeks after an uneventful pregnancy. In addition to small for gestational age (weight 1900 g, -3.6 SDS; length 43 cm, -4.1 SDS; head circumference 33.0 cm, -1.3 SDS) and relative macrocephaly at birth, no other abnormality was noted. The patient had severe feeding difficulties during neonatal period but without the need of feeding tube. No significant lag in psychomotor development was found. Echocardiography revealed atrial septal defect when he was 4 years old, and hence, he had undertaken surgical treatment. He firstly visited our hospital due to growth retardation (length 94 cm, -3.3 SDS, weight 11.5 kg, -3.3 SDS) at 4.5 years old (**Figure 1A**) (Zhang et al., 2017). Clinical findings (**Table 1** and **Figure 1B**) of him were similar to that of previously reported (Begemann et al., 2015). Facial dysmorphisms included triangular face, micrognathia, and low-set ears. He had ambiguous genitalia with a small penis and hydrocele. Asymmetrical body, hands, and feet were obvious. He presented slight hypotonia and a high-pitched voice. Differential diagnosis excluded the existence of congenital infections, mitochondrial diseases, and metabolic diseases. Accordingly, he started growth hormone (GH) therapy from 4.9 years of age and it was efficacious in the promotion of growth, however, endocrinological investigation was still abnormal. Serum IGF1 at 11 years old was 151.6 ng/ml (-2 SDS) and 13 years old was 319 ng/ml (-2 SDS). Insulin-like growth factor-binding protein 3 at 11 years old was 3.63 μg/ml (-2 SDS). The detection method and reference values of serum IGF-1 and IGFBP-3 levels were strictly according to that previously reported (Xu et al., 2010). Based on the clinical symptoms and Netchine–Harbison scoring system (Azzi et al., 2015), he was diagnosed as SRS. This study was approved by the Medical Ethics Committee in The Second Hospital of Anhui Medical University. All participants have provided written informed consents and that the subject’s parents provided written consent to publish the report.

**Table 1:** Clinical features in patients with growth restriction who had mutations in IGF2.

		Begemann et al., 2015				Yamoto et al., 2017
	Present case	Patient 1	Patient 2	Patient 3	Patient 4	Japanese case

		**General characteristics**				
**Gender**	**M**	**M**	**F**	**M**	**F**	**M**

**Country of origin**	**China**	**Germany**				**Japan**

**Clinical features**				
Short stature	+	+	+	+	+	+
Relative macrocephaly at birth	+	+	+	+	+	+
Frontal bossing	+	+	+	-	+	+
Low set ears in childhood	+	+	+	+	+	+
Triangular face	+	+	+	+	+	+
Body asymmetry	+	-	-	-	-	-
Reduced stamina	+	+	+	+	NA	NA
Pigment nevi	Several	Several	Many	Several	Several	NA
Finger or toe deformities	+	+	+	-	+	+
High-pitched voice in childhood	+	+	+	+	+	NA
Micrognathia or retrognathia	+	+	+	-	+	+
Ambiguous genitalia	+	NA	NA	+	NA	+
Heart	Atrial septal defect (taken surgical treatment at 4 years old)	Persistent ductus arteriosus, ventricular septum defect, no surgical intervention	Persistent ductus arteriosus, spontaneous closure	-	Small ventricular septum defect	Pulmonary hypertension
Response to growth hormone in the first year	+	+	+	+	-	NA

## *Igf2* Variant as Candidate for Pathopoiesis

**FIGURE 2:**
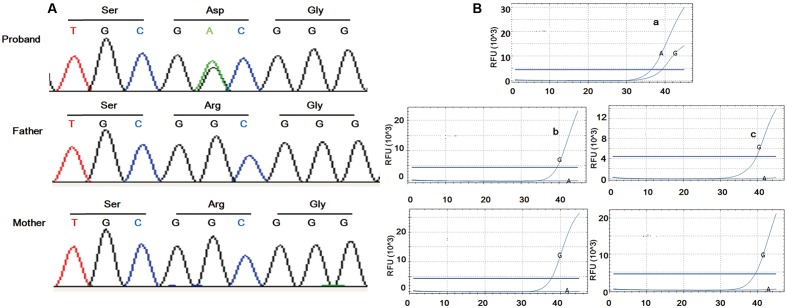
Sanger sequencing and ARMS-PCR results. Sanger sequencing of the family trio illustrated *de novo* mutation in the patient **(A)**. Amplification of the proband was found using the mutational primers **(Ba)** while amplification was implemented using wild type primers in the controls **(Bb–e)**.

In order to identify the molecular pathogenesis, auxiliary examination was performed. Chromosome analysis showed a 46, XY karyotype. ICR1 hypomethylation analysis was negative. Low coverage whole-genome sequencing did not detect clinically significant abnormal CNVs and maternal uniparental disomy 7 (isodisomic). Maternal uniparental disomy 7 (heteroisomy) was not excluded due to limited experimental condition. Whole exome sequencing (WES) was applied to the family trio. Detailed method was shown in Supplementary 1. The percentage of coverage and average depth for WES were 99.76% and 118.56X. No *CDKN1C* mutation was found (coverage > 10X, 99%; coverage > 20X, 90%; depth of the gene: min/max/mean, 7X/238X/106.54X). As the serum IGF1 was decreased, we checked the sequencing data of IGF1 and IGFR. The results showed that they were both normal (the depth of IGF1 and IGF1R were 124.48X and 237.49X), however, a heterozygous variant in *IGF2* (NM_000612, c.101G > A, p.Gly34Asp) was identified. The same site of the parents was wild type. Sanger sequencing confirmed the result of WES (**Figure 2A**). This variant was not recorded in any of the publicly available SNP database (dpSNP, 1000Genomes, ExAC, ESP, HGMD, Clinvar) and damage prediction showed that it was harmful. According to ACMG guideline (Richards et al., 2015), the interpretation of the variant was PS2+PM2+PP1+PP3+PP4+PP5, which, met the standard of “pathogenic.” Together, it is possible to assume that *IGF2* might be the candidate pathogenic gene.

## The Mutation Site Locateed on the Paternal Allele

As IGF2 is documented to be paternally expressed in most tissues (DeChiara et al., 1991) and monoallelically expressed in whole peripheral blood leukocytes (Frost et al., 2010), to identify whether the mutation was on the paternal allele, RNA from peripheral blood was used for amplification refractory mutation system polymerase chain reaction (ARMS-PCR). Fluorescence probe method (Supplementary 2) was applied to increase binding specificity. Significant amplification for the proband was found using the mutational primers, whereas amplification was implemented using wild type primers in healthy controls (**Figure 2B**). The result indicated that the mutation locates on the paternal allele.

## Structural Modeling Results

**FIGURE 3:**
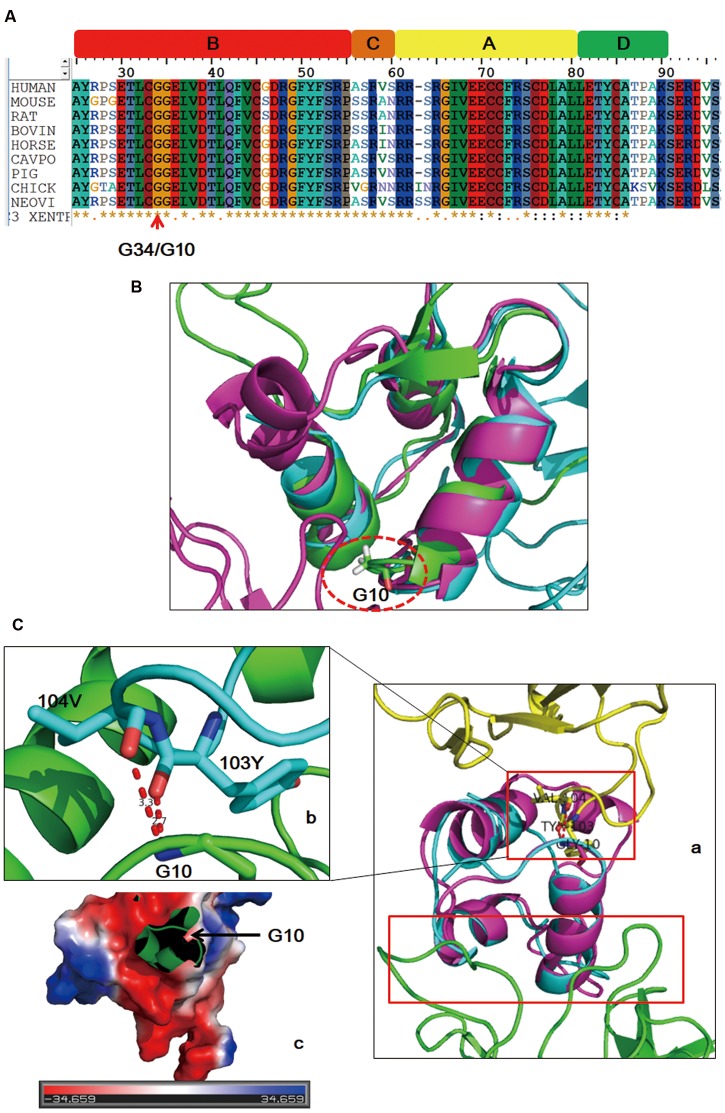
Sequence alignment and structure analysis results. IGF2 is evolutionarily conserved across species **(A)**. The mutated site (dashed circle) is in adjacent to the first α helix of IGF2 in both free and binding states **(B)**. Schematic drawing of the binding sites (red boxes) of IGF2 with its two different binding partners (**Ca**: yellow, IGF2R; green, IGF2 antibody). Detailed interaction of G10 with its binding sites **(Cb)**. Binding surface of the local region on IGF2 around G10 is negatively charged **(Cc)**.

To further investigate possible pathogenicity of this mutation for the onset of the disease, sequence alignment, and structural modeling were performed. The whole sequence including the identified site of IGF2 is evolutionarily conserved across species (**Figure 3A**). Generally, amino acids with high conservation are considered to be sensitive to mutation, and thus, the identified site is likely to be the etiology. Online IGF2 apo structure (PDB 1IGL) and structures of IGF2 in complex with its binding partners (PDB 2V5P, 3KR3) were enrolled. The identified site is G10 in mature IGF2 and G10 is adjacent to the first α helix in all structures (**Figure 3B**), indicating that the local structure of this region has strong rigidity. The first α helix is involved in the binding of IGF2 with partners (**Figure 3Ca**). For complex IGF2/IGF2R (2V5P), G10 is involved in the interaction (**Figure 3Cb**) and the variant brings bout a long side chain which will induce steric hindrance. In addition, IGF2 has an overall negatively charged surface at the binding site (**Figure 3Cc**), and G10D variant will change local charge state. In light of this, it is possible that G10D variant might affect the binding of IGF2 with partners by direct interaction disruption or surface charge modification.

## Discussion

Silver–Russell syndrome brings about various forms of unusual physical characteristics and functional defects, resulting in the clinical diagnosis difficult. In 2016, the international consensus statement on SRS has been produced (Wakeling et al., 2017), which, has particular guiding significance for clinicians. Netchine–Harbison clinical scoring system (Azzi et al., 2015) was recommended by the consensus statement. Our patient was found with (small for gestational age) SGA, relative macrocephaly at birth, feeding difficulties, growth retardation, body asymmetry, and micrognathia, which, met the clinical diagnostic criteria. Body asymmetry identified here is a distinguishing feature not reported previously in patients with IGF2 mutation.

Currently, positive molecular investigation is reported in about 60% patients with SRS (Netchine et al., 2007). Notwithstanding molecular testing is negative in a notable proportion of patients who are suspected as SRS, molecular diagnosis plays important roles for the diagnosis of patients with few or atypical features and the management of SRS. For example, GH therapeutic effect varies according to the underlying syndromic diagnosis (Verge et al., 1994; Renes et al., 2013; Wakeling et al., 2017). To further define the type of mutation and provide more tailored management, we firstly examined 11p15 methylation pattern as it is the mostly documented molecular pathology (Schonherr et al., 2006). As the result was normal, next generation sequencing including WES and low-coverage whole genome sequencing were performed. Clinically significant CNVs, maternal uniparental disomy 7 (isodisomic), and *CDKN1C* variants were not detected, however, a *de novo* heterozygous *IGF2* c.101G > A (p.Gly34Asp) variant was identified. Sanger sequencing showed it was *de novo*. The variant was pathogenic according to ACMG guideline criteria (Richards et al., 2015).

IGF2 is documented to be linked to growth retardation, overgrowth, obesity, polycystic ovary syndrome, and cancer (Livingstone, 2013). IGF2 induced growth retardation is reported to be linked to a paternally methylated imprinting control region (Demars and Gicquel, 2012). Begemann et al. (2015) firstly reported growth restriction caused by *IGF2* mutation. Accordingly, the identified *IGF2* variant was suspected as causal pathogenesis of the proband. It is reported that the paternal allele of *IGF2* is transcribed while the maternal allele is silenced (DeChiara et al., 1991), therefore we hypothesized that the mutation located on the paternal allele. ARMS-PCR validated our assumption and further illustrated the possibility of the identified variant as the pathogenic.

IGF2 is the predominant IGF in adults and mainly exists in the form of complex (Baxter et al., 1995). Freely circulated IGF2 is unstable and subject to degradation, hence, complex state is important for IGF2 function. IGF2 combines with a family of six insulin-like growth factor-binding proteins and receptors including IGF2 receptor, IGF1 receptor, and insulin receptor to mediate cell proliferation, differentiation, migration, and survival. In line with this, conformational change of IGF2 is important for its physiological regulation. Structural modeling showed that the mutated site influence the binding of IGF2 with its objects, which might in turn accelerate the degradation of IGF2.

To date, patients with growth retardation from a multigenerational family with *IGF2* point mutation and a sporadic case of *de novo IGF2* indel mutation were reported (Begemann et al., 2015; Yamoto et al., 2017). Herein, we present a sporadic case with *de novo* mutation in *IGF2.* To our knowledge, this is only the second report of a pathogenic *de novo IGF2* variant. We suggest that investigating for an IGF2 mutation could be considered when investigating individuals with an SRS phenotype; we acknowledge that it is likely that mutations in this gene will likely explain the etiology of SRS in a minority of individuals. Further reports will be critical to refining the molecular and clinical features, including the pattern of GH responsiveness, associated with IGF2 mutations.

## Author Contributions

DgL was an attending pediatrician, collected clinical information. YW drafted the initial manuscript. X-AY conceptualized and designed the study, performed data analysis, and wrote the manuscript. DyL designed the study, critically reviewed the manuscript. All authors approved the final manuscript as submitted and agree to be accountable for all aspects of the work.

## Conflict of Interest Statement

YW was employed by company Joy Orient Translational Medicine Research Center Co. Ltd., and X-AY was employed by company Beijing Scientific Operation Biotechnology Co. Ltd. The other authors declare that the research was conducted in the absence of any commercial or financial relationships that could be construed as a potential conflict of interest.
