# Supplementary material for: De Novo Mutation of Paternal IGF2 Gene Causing Silver–Russell Syndrome in a Sporadic Patient
Source: Front Genet. 2017 Aug 8;8:105. doi: 10.3389/fgene.2017.00105 (PMC5550680; doi:10.3389/fgene.2017.00105)
Supplement: Supplementary file 1 [file Data_Sheet_1.DOCX]

**Supplementary 1 Gene sequencing.**

**Next generation sequencing (NGS) and data analysis.**

NGS was performed using Illumina Hiseq2500 (Illumina, Santiago, USA). Briefly, genomic DNA was extracted from leucocyte of 2~4 ml peripheral blood using BloodGen Midi Kit (CWBIO, China), and was then hybridized and enriched for whole exome sequencing (WES) and low-coverage whole genome sequencing strictly according to the manufactures’ protocol. Libraries were captured using SureSelect Human All Exon V6 (Agilent Technologies) and were applied to Illumina Hiseq2500. Raw image files were processed and the sequencing reads were aligned to the human reference genome (hg19) using Burrows–Wheeler Alignment (BWA). GATK (McKenna et al. 2010) and Annovar (Wang et al. 2010) were used to analyze the candidate variants. A 0.5% frequency cut-off of 1000 genomes project and Exac database was applied. For low-coverage whole genome sequencing data, CNVs of 100 kb or larger were analyzed using self-developed software (Joyorient, Beijing, China) and then measured with the database of (Decipher, ClinVar, HGMD and OMIM). Non-synonymous, loss-of-function, indel, duplication, splice site variants, and copy number variants (CNVs) were taken for candidate variants identification.

**Sanger sequencing**

For Sanger sequencing, the primers used were: IGF2-F, 5’- CTTTTCTGTGTGCTTCTGGACGCTT-3’ and IGF2-R, 5’- GCACGTCCCTTGTCAGAATGTCCAA-3’, harboring a product of 788 bp in length. The program was as follows: 95 ℃ for 4 min, followed by 30 cycles of denaturation at 95 ℃ for 30 s, and annealing/extension temperature of (60/72) for 1min, and a final extension of 72 for 10 min. The products were further sequenced with ABI 3730XL (Thermo Fisher Scientific, Waltham, MA, USA).

**Supplementary 2 Amplification refractory mutation system polymerase chain reaction (ARMS-PCR).**

Total RNA was extracted with Trizol (sigma, St.Louis, USA) from was extracted from leucocyte of 2~4 ml peripheral blood from the patient and 4 healthy control. Total mRNA reverse transcription reaction was performed using PolyA plus tail method. The synthesized cDNA used as the template and ABI 7500 (Thermo Fisher Scientific, Waltham, MA, USA) was used for PCR amplification. The sequences of primers used were as follows: IGF2-FG, 5’- CAGTGAGACCCTGTGCGG-3’; IGF2-FA, 5’- CAGTGAGACCCTGTGCGA-3’; IGF2-R, 5’- ACAGCACTCCTCAACGATGC-3’, and fluorescence probe 5’-AGCTGGTGGACACCCTCCAGTTCG-3’. The result of amplification was illustrated in the following table.

**Ct value and genotyping**

| Sample | IGF2-FG Ct value | IGF2-FA Ct value | Genotyping |
| --- | --- | --- | --- |
| Patient | 39.17 | 36.4 | A |
| Control 1 | 39.43 | NA | G |
| Control 2 | 39.73 | NA | G |
| Control 3 | 37.54 | NA | G |
| Control 4 | 39.29 | NA | G |

Note: NA, not observed.
